# Complementary cardiac functional assessment using systolic time intervals and bioelectrical impedance analysis

**DOI:** 10.1136/openhrt-2026-004041

**Published:** 2026-06-08

**Authors:** Tomoaki Nishikawa, Akinori Higaki, Keisho Kurokawa, Arisa Abe, Rikako Horie, Ryo Miyabe, Yasuhisa Nakao, Tomoki Fujisawa, Yusuke Akazawa, Toru Miyoshi, Hiroshi Kawakami, Haruhiko Higashi, Shunsuke Tamaki, Kazuhisa Nishimura, Katsuji Inoue, Shuntaro Ikeda, Osamu Yamaguchi

**Affiliations:** 1 Department of Cardiology, Pulmonology, Hypertension & Nephrology, Ehime University Graduate School of Medicine, Toon, Japan

**Keywords:** HEART FAILURE, Atherosclerosis, Risk Factors, Echocardiography

## Abstract

**Background:**

The Forrester classification remains a cornerstone for assessing haemodynamic status in heart failure, yet its clinical application is restricted by the invasiveness of right heart catheterisation. This study aimed to develop a novel, non-invasive and reproducible alternative by integrating bioelectrical impedance analysis (BIA) and pulse wave analysis (PWA).

**Method:**

We conducted a retrospective study of consecutive patients admitted to our cardiology department between January 2020 and December 2022 who underwent PWA within 1 month of BIA. Systolic time intervals (STIs) were calculated as the ratio of pre-ejection period to left ventricular ejection time using PWA. Patients were classified into four virtual Forrester profiles—*Warm and Dry*, *Warm and Wet*, *Cold and Dry*, and *Cold and Wet*—based on STI and extracellular water/total body water thresholds. The primary endpoint was major adverse cardiovascular events (MACEs).

**Results:**

Among 558 patients with available STI and BIA data, 161, 209, 90 and 98 were classified as *Warm and Dry*, *Warm and Wet*, *Cold and Dry*, and *Cold and Wet*, respectively. *Wet* groups were older, had higher B-type natriuretic peptide, lower skeletal muscle mass and haemoglobin and more renal impairment, whereas *Cold* groups exhibited lower left ventricular ejection fraction. During follow-up, 84 MACEs occurred. Kaplan-Meier analysis showed significant differences in MACE-free survival among the four profiles (log-rank p=0.01), with *Warm and Dry* having the most favourable prognosis.

**Conclusion:**

The proposed virtual Forrester profiling integrating BIA and PWA provides a practical, quantitative and reproducible framework for non-invasive haemodynamic assessment.

**WHAT IS ALREADY KNOWN ON THIS TOPIC**

- While the Forrester classification is fundamental for haemodynamic profiling, its clinical use is limited by the need for invasive right heart catheterisation, and current non-invasive surrogates like echocardiography often lack objectivity or suffer from significant inter-observer variability.

**WHAT THIS STUDY ADDS**

- Integrating bioelectrical impedance analysis and pulse wave analysis allows for a ‘Virtual Forrester Profiling’ that objectively categorises patients into four subsets with distinct clinical characteristics and prognostic risks for major adverse cardiovascular events.

**HOW THIS STUDY MIGHT AFFECT RESEARCH, PRACTICE OR POLICY**

- This quantitative and easily reproducible method offers a practical, time-efficient alternative for non-invasive haemodynamic monitoring and preoperative risk assessment, particularly in clinical settings where specialised resources are limited.

## Background

 The Forrester classification is a haemodynamic profiling system developed to assess the severity and underlying pathophysiology of acute heart failure, first introduced by Forrester in the 1970s.^1^ This system stratifies patients into four distinct subsets based on measurements of cardiac index (CI) and pulmonary artery wedge pressure (PAWP), with each profile reflecting specific circulatory conditions that assist in guiding prognosis and therapeutic strategies. Although the clinical utility of this classification is beyond dispute, a well-known limitation is its reliance on invasive data acquisition via right heart catheterisation. Consequently, alternative approaches have been proposed and implemented in clinical practice, such as the Nohria-Stevenson classification based on physical examination findings,^2^ and surrogate methods using echocardiographic parameters.^3 4^ However, the former approach is limited by a lack of objectivity and quantifiability, while the latter, echocardiography-based methods, have yet to undergo sufficient validation. Therefore, we devised a novel non-invasive alternative to the Forrester classification by integrating two types of physiological testing methods other than echocardiography, aiming to ensure objectivity, quantifiability, simplicity and reproducibility.

As a substitute for PAWP, we focused on bioelectrical impedance analysis (BIA). BIA is a technique that quantitatively assesses body composition by measuring the impedance generated when a weak electrical current is passed through the body. Previous studies have reported that the extracellular water to total body water ratio (ECW/TBW) derived from BIA serves as a diagnostic indicator of congestive heart failure, a marker of therapeutic responsiveness, and shows a significant positive correlation with B-type natriuretic peptide (BNP) levels.^5^,^7^ More recently, Katz *et al* reported that, in patients with congenital heart disease, those with an ECW/TBW >0.39 exhibited significantly higher PAWP compared with individuals with lower ECW/TBW.^8^ We also identified an ECW/TBW >0.394 as the optimal cut-off value to detect elevated BNP levels (>200 pg/mL).^9^ For a non-invasive surrogate of CI, we considered the systolic time interval (STI) obtained from pulse wave analysis (PWA) as a candidate. STI is calculated as the ratio of the pre-ejection period (PEP) to the left ventricular ejection time (LVET), a parameter known to correlate with myocardial contractility.^10^ Indeed, prior studies have demonstrated a strong correlation between invasively measured stroke volume and PEP/LVET.^10 11^ Thus, by constructing a matrix with ECW/TBW on the horizontal axis and STI on the vertical axis, it becomes possible to classify patients into four haemodynamic subsets analogous to the Forrester and Nohria-Stevenson classifications; a concept we term ‘Virtual Forrester Profiling’. The present study is a pilot investigation aimed at establishing proof-of-concept for this novel classification method.

## Methods

### Study population

We conducted a study involving consecutive patients admitted to our cardiology department from 1 January 2020 to 31 December 2022. Among these, only patients who underwent PWA within 1 month of BIA were included in the analysis. We collected relevant clinical variables, including Hb, haematocrit, BNP and estimated glomerular filtration rate (eGFR), within 1 day of the BIA. The primary endpoint of this study was the occurrence of major adverse cardiovascular events (MACEs). In this study, MACE was defined as a composite of all-cause death, cerebrovascular events requiring hospitalisation, hospitalisation for worsening heart failure, acute coronary syndrome and unexpected coronary revascularisation. Event data were collected through a review of electronic medical records, and the occurrence of each endpoint was confirmed up to 31 December 2024.

### Bioelectrical impedance analysis

A BIA was performed once during hospitalisation using the InBody770 (InBody Japan, Tokyo, Japan) for all patients whenever possible as previously described.^7^ Specifically, for patients in stable condition admitted for scheduled hospitalisation, a BIA was conducted on the day of admission. For those admitted in a critical condition, a BIA was performed as soon as their condition stabilised, they were transferred to the general ward and were able to stand unassisted.

### Measurement of STIs

STI were measured by trained technicians using a PWA testing device (FORM-5, Fukuda Colin, Tokyo, Japan). On the mechanocardiogram, the total electromechanical systolic interval (Q-II) was defined as the time from the onset of ventricular depolarisation (Q wave on ECG) to the first high-frequency component of the aortic component of the second heart sound (S2) on the phonocardiogram (PCG). The LVET was defined as the duration from the upstroke to the dicrotic notch of the brachial arterial waveform measured by plethysmography. The PEP was calculated by subtracting LVET from Q-S2, and the STI was defined as the ratio of PEP to LVET (PEP/LVET).^10^

### Definition of virtual Forrester profile

**Figure 1:**
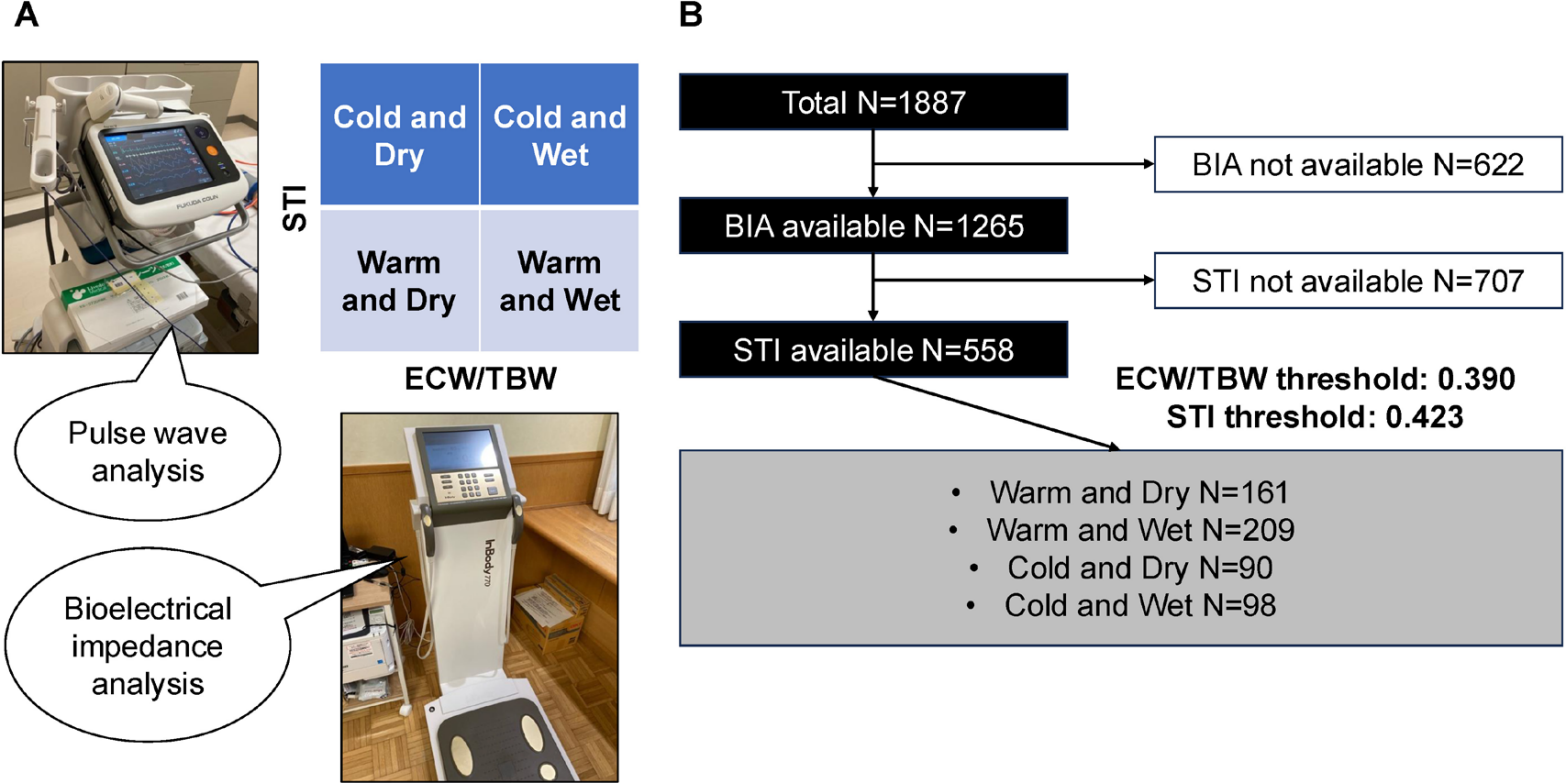
Concept of virtual Forrester profiling and patient enrolment flow chart. (**A**) Schematic of virtual Forrester profiling integrating bioelectrical impedance analysis (BIA) and pulse wave analysis (PWA). Systolic time intervals (STIs) derived from PWA reflect cardiac contractility, whereas the extracellular water to total body water ratio (ECW/TBW) derived from BIA reflects volume status. Patients are classified into four haemodynamic profiles—Warm and Dry, Warm and Wet, Cold and Dry, and Cold and Wet—based on STI and ECW/TBW thresholds. (**B**) Flow chart of patient selection. Among 1887 hospitalised patients, 1265 underwent BIA, of whom 558 had paired STI and BIA data available for virtual Forrester profiling and subsequent analysis.

Patients were classified into four groups based on the presence or absence of systolic dysfunction and cardiac congestion (figure 1A). Subjects with an STI above the threshold were categorised as ‘Cold’, whereas those with a lower STI were defined as ‘Warm’. Similarly, subjects with an ECW/TBW ratio above the threshold were classified as ‘Wet’, and those below the threshold were defined as ‘Dry’. Consequently, the entire cohort was divided into four categories: *Warm and Dry*, *Warm and Wet*, *Cold and Dry*, and *Cold and Wet*. The thresholds for STI and ECW/TBW were set at 0.423 and 0.390, respectively, based on previous literature.^8 9 12 13^

### Statistical analysis

Statistical analyses were performed using R (V.4.4.1; Foundation for Statistical Computing, Vienna, Austria). Continuous variables are expressed as medians (IQRs). The significance of differences was assessed using Welch’s t-test. Categorical variables are presented as numbers and percentages, and group differences were evaluated using the χ^2^ test. A one-way analysis of variance followed by post hoc Bonferroni correction was applied for multiple group comparisons. The correlation between parameters was assessed using Spearman’s correlation coefficient. A correlation coefficient (Rho) above 0.5 was interpreted as indicating a strong correlation.

Survival among the four categories was estimated using the Kaplan-Meier method, and differences in survival curves were evaluated by the log-rank test.

## Results

### Patient characteristics

Figure 1B shows the flowchart of patient enrolment. Among 1887 hospitalised patients, BIA was performed in 1265. Among the population, STI was available in 558 subjects. Regarding the timing of PWA and BIA, a post hoc analysis revealed that the measurements were performed concurrently in most cases: 76.3% (n=426) were on the same day and 4.1% (n=23) were within 1 day, with an overall average interval of 1.38±4.7 days. Based on our definition, the number of patients belonging to *Warm and Dry*, *Warm and Wet*, *Cold and Dry*, and *Cold and Wet* were 161, 209, 90 and 98, respectively.

The baseline characteristics of patients stratified by the virtual Forrester profile are summarised in table 1. Patients in the *Warm and Wet* and *Cold and Wet* groups were significantly older than those in the *Warm and Dry* and *Cold and Dry* groups (median age: 77.0 and 72.5 years vs 61.0 and 56.0 years, p<0.01). The proportion of male patients was highest in the *Warm and Dry* and *Cold and Dry* groups (70.2% and 70.0%, respectively), while it was lowest in the *Warm and Wet* group (51.7%, p<0.01). No significant differences were observed in body mass index across the four groups. However, the *Wet* groups exhibited significantly higher ECW/TBW ratios than the *Dry* groups (both 0.40 vs 0.38, p<0.01). Conversely, the skeletal muscle mass index was lower in the *Wet* groups, suggesting poorer nutritional or physical status (p<0.01). Regarding haemodynamic indices derived from PWA, patients in the *Cold* groups showed prolonged PEP and shortened LVET, resulting in markedly elevated STI compared with those in the *Warm* groups (p<0.01 for all comparisons). The cardiothoracic ratio was also higher in the *Wet* groups (p<0.01). Left ventricular ejection fraction (LVEF) was significantly reduced in the *Cold* groups (median 52.3% and 50.0%) compared with the *Warm* groups (63.2% and 63.8%, p<0.01). Laboratory findings showed that haemoglobin and eGFR were lower in the *Wet* groups, consistent with more advanced systemic congestion or renal impairment (p<0.01). BNP levels were significantly elevated along the ‘Wet’ axis, being lowest in the *Warm and Dry* group (median=11.0 pg/mL) and highest in the *Cold and Wet* group (median=111.3 pg/mL, p<0.01). The distribution of underlying diseases also differed markedly among the groups (p<0.01). Chronic coronary syndrome was most prevalent in the *Warm and Wet* group (54.1%), while cardiomyopathy was more frequent in the *Cold and Dry* group (20.0%). Heart failure and valvular disease were particularly common in the *Cold and Wet* group (14.3% and 6.1%, respectively), whereas tachyarrhythmia was the dominant diagnosis in the *Warm and Dry* group (44.1%).

**Table 1:** Patient characteristics according to the virtual Forrester profiling

	Warm and dry (n=161)	Warm and wet (n=209)	Cold and dry (n=90)	Cold and wet (n=98)	P value
Age	61.00 (48.00, 72.00)	77.00 (70.00, 81.00)	56.00 (44.00, 67.00)	72.50 (66.25, 75.00)	<0.001
Male	113 (70.2)	108 (51.7)	63 (70.0)	65 (66.3)	0.001
BMI (kg/m^2^)	23.54 (21.75, 25.73)	22.93 (20.25, 25.47)	23.53 (21.89, 26.11)	23.26 (20.79, 25.76)	0.219
ECW/TBW	0.38 (0.38, 0.39)	0.40 (0.39, 0.40)	0.38 (0.38, 0.39)	0.40 (0.39, 0.40)	<0.001
Skeletal muscle mass index (kg/m^2^)	7.20 (6.40, 7.70)	6.40 (5.70, 7.30)	7.30 (6.32, 7.90)	6.75 (5.90, 7.68)	<0.001
Body fat percentage	0.27 (0.23, 0.32)	0.29 (0.23, 0.35)	0.27 (0.22, 0.31)	0.28 (0.22, 0.36)	0.056
PEP (ms)	103.00 (96.00, 111.00)	104.00 (94.00, 113.00)	129.50 (120.00, 146.00)	128.50 (122.00, 138.75)	<0.001
LVET (ms)	294.00 (279.00, 309.00)	306.00 (287.00, 328.00)	264.00 (252.25, 274.75)	265.00 (247.25, 279.50)	<0.001
Systolic time intervals	0.35 (0.32, 0.39)	0.34 (0.30, 0.38)	0.48 (0.44, 0.56)	0.47 (0.44, 0.53)	<0.001
Cardiothoracic ratio (%)	46.80 (43.38, 49.60)	51.20 (47.50, 54.60)	49.45 (46.42, 52.50)	53.35 (49.62, 58.70)	<0.001
Sinus rhythm	158 (98.1)	187 (89.5)	85 (94.4)	73 (74.5)	<0.001
LVEF (%)	63.20 (58.73, 67.55)	63.80 (58.20, 68.53)	52.30 (34.05, 60.15)	50.00 (40.00, 59.40)	<0.001
Haemoglobin (g/dL)	14.00 (13.30, 15.00)	12.70 (11.50, 13.70)	14.55 (13.60, 15.50)	13.35 (11.93, 14.40)	<0.001
eGFR (mL/min/1.73 m^2^)	72.40 (62.10, 84.10)	56.90 (42.40, 69.40)	70.40 (56.57, 84.08)	58.45 (43.50, 72.35)	<0.001
BNP (pg/mL)	11.00 (6.30, 22.85)	47.00 (22.25, 88.10)	20.80 (8.60, 67.40)	111.25 (46.72, 277.08)	<0.001
Disease categories					<0.001
ACHD	8 (5.0)	2 (1.0)	8 (8.9)	2 (2.0)	
ACS	9 (5.6)	9 (4.3)	2 (2.2)	7 (7.1)	
Brady-arrhythmia	0 (0.0)	11 (5.3)	1 (1.1)	3 (3.1)	
Cardiomyopathy	3 (1.9)	2 (1.0)	18 (20.0)	9 (9.2)	
CCS	62 (38.5)	113 (54.1)	25 (27.8)	33 (33.7)	
Heart failure	1 (0.6)	4 (1.9)	5 (5.6)	14 (14.3)	
IE	0 (0.0)	1 (0.5)	0 (0.0)	0 (0.0)	
PAD	2 (1.2)	4 (1.9)	1 (1.1)	1 (1.0)	
Pericardial disease	0 (0.0)	4 (1.9)	0 (0.0)	0 (0.0)	
Pulmonary artery disease	1 (0.6)	9 (4.3)	1 (1.1)	1 (1.0)	
Tachy-arrhythmia	71 (44.1)	32 (15.3)	25 (27.8)	20 (20.4)	
Valvular disease	4 (2.5)	16 (7.7)	4 (4.4)	6 (6.1)	
Others	0 (0.0)	2 (1.0)	0 (0.0)	2 (2.0)	

ACHD, adult congenital heart disease; ACS, acute coronary syndrome; BMI, body mass index; BNP, B-type natriuretic peptide; CCS, chronic coronary syndrome; ECW/TBW, extracellular water/total body water; eGFR, estimated glomerular filtration rate; IE, infectious endocarditis; LVEF, left ventricular ejection fraction; LVET, left ventricular ejection time; PAD, peripheral artery disease; PEP, pre-ejection period.

### Correlation between clinical parameters and the distribution of the patients

**Figure 2:**
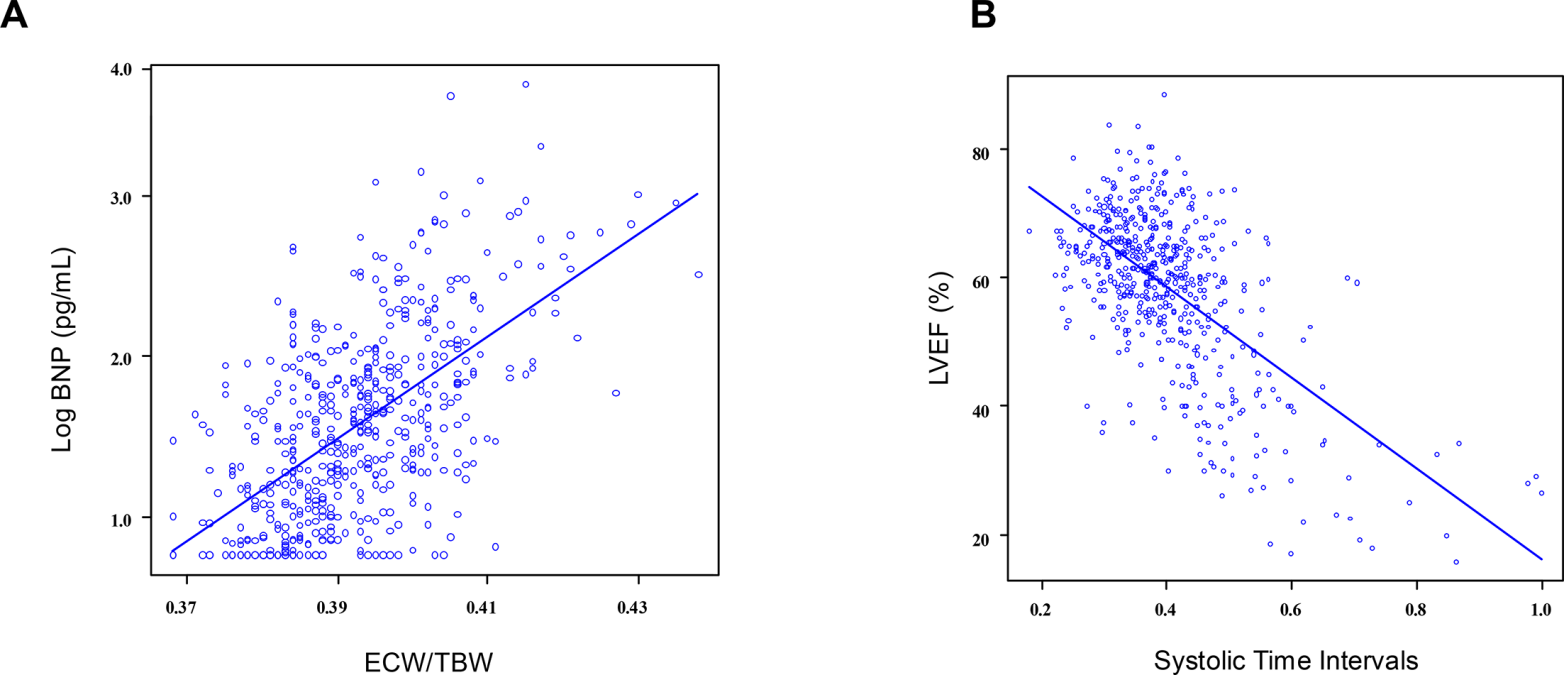
Correlations between non-invasive clinical parameters and cardiac biomarkers/function. (**A**) Scatter plot showing a significant positive correlation between extracellular water to total body water ratio (ECW/TBW) and log-transformed brain natriuretic peptide (Log BNP) (rho=0.57, p<0.01). (**B**) Scatter plot showing a significant negative correlation between systolic time intervals (STIs) and left ventricular ejection fraction (LVEF) (rho=0.48, p<0.01). The solid lines represent the linear regression trends.

The correlation coefficient between BNP and ECW/TBW was 0.57 (p<0.01) and the coefficient between LVEF and STI was −0.48 (p<0.01) (figure 2).

**Figure 3:**
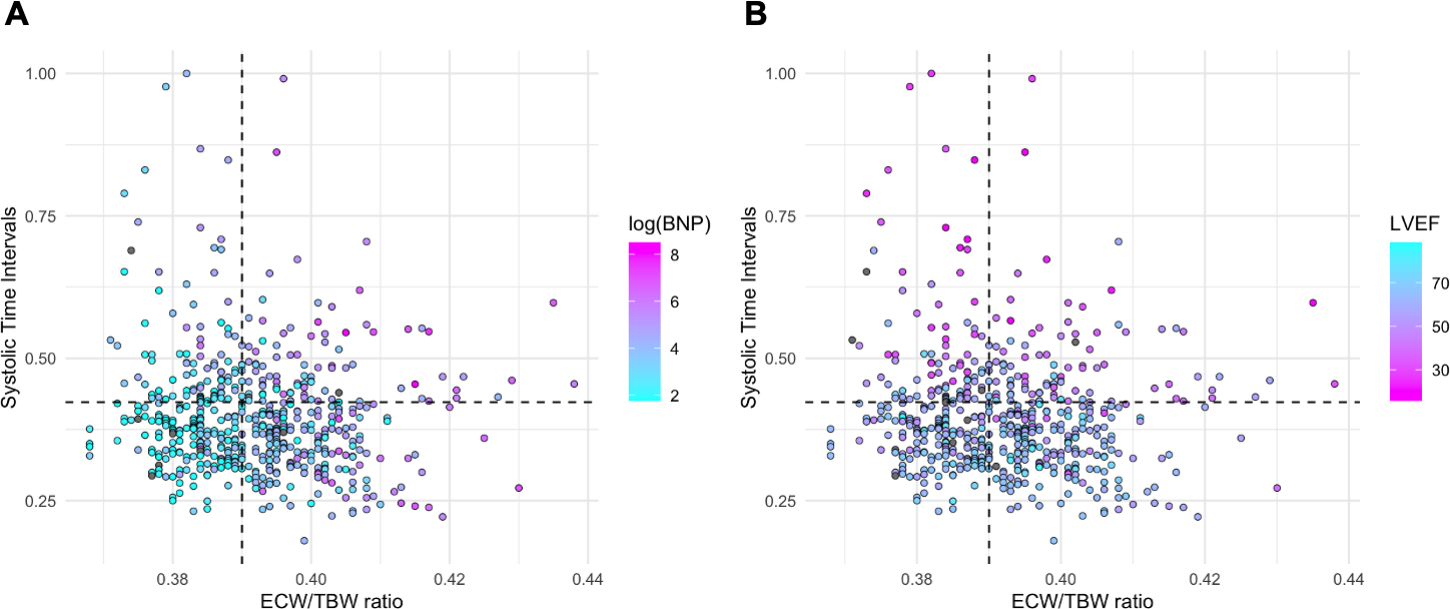
Distribution of patients in the STI–ECW/TBW matrix. Patients are plotted by systolic time intervals (STIs) and extracellular water/total body water ratio (ECW/TBW). (**A**) Scatter plot colour-coded by log(BNP), showing higher values on the right side of the plot. (**B**) Scatter plot colour-coded by LVEF, showing lower values towards the top. Dashed lines indicate thresholds for the four virtual Forrester profiles: Warm and Dry, Warm and Wet, Cold and Dry, and Cold and Wet. BNP, B-type natriuretic peptide; LVEF. left ventricular ejection fraction.

The patients’ clinical data were mapped with colour on the scatter plot by STI and ECW/TBW. As shown in figure 3A, data points with higher log (BNP) were distributed on the right side of the plot, while low EF data points were distributed on the top side of the plot as shown in figure 3B. There was quite weak negative correlation between ECW/TBW and STI (Rho=−0.11, p=0.01).

### Survival analysis

**Figure 4:**
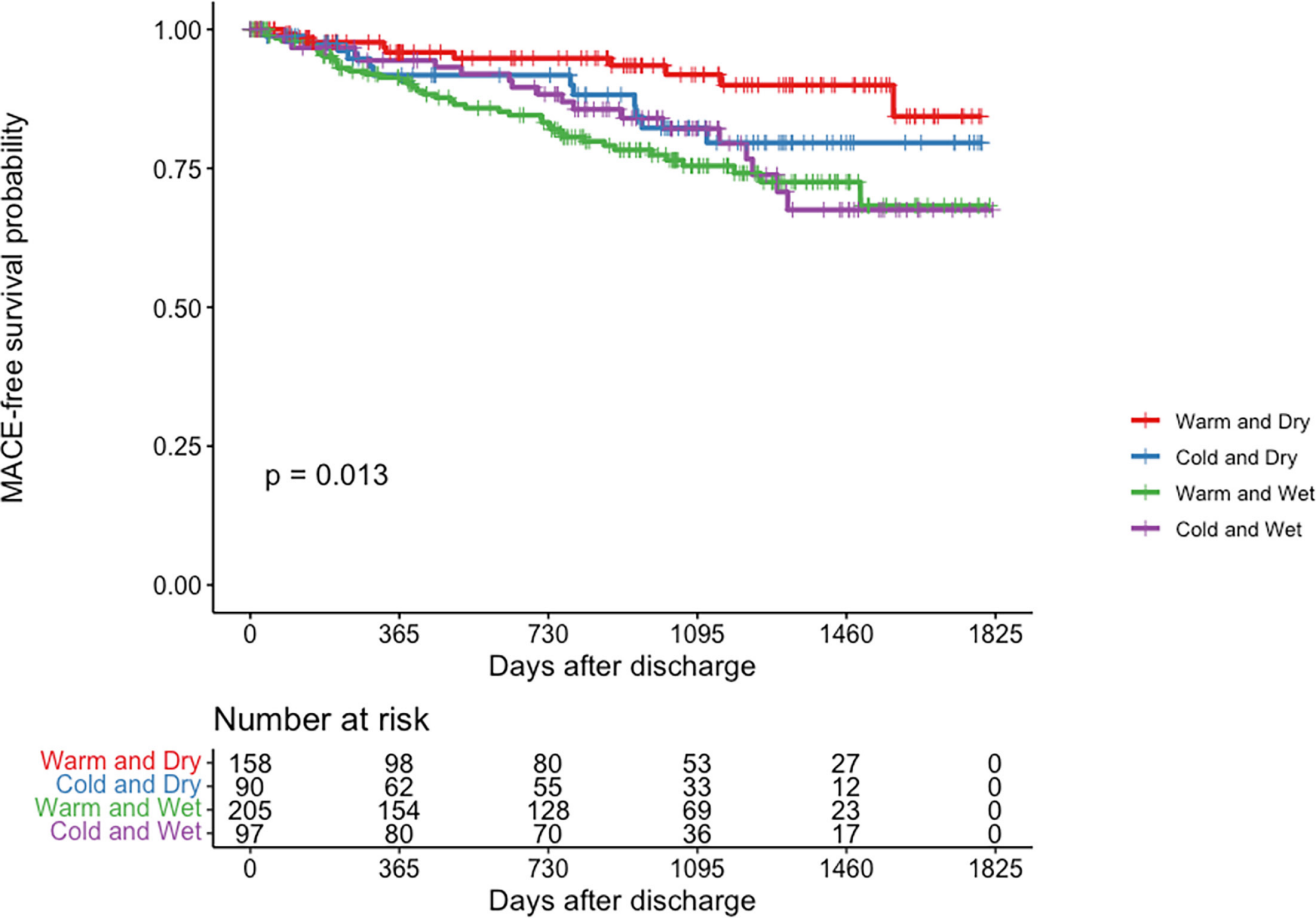
Kaplan-Meier analysis of major adverse cardiovascular events (MACEs) according to virtual Forrester profiles. Survival curves for MACE-free survival stratified by virtual Forrester profiles in the study cohort (n=558). The Warm and Dry group exhibited the highest event-free survival, whereas the Cold and Wet group had the lowest. Differences among groups were statistically significant (log-rank p=0.01).

During the follow-up period of up to 5 years after discharge, a total of 84 MACEs occurred. Kaplan-Meier analysis shown in figure 4 revealed significant differences in MACE-free survival among the four groups (log-rank p=0.01). The *Warm and Dry* group demonstrated the most favourable prognosis, whereas the *Cold and Wet* group showed the poorest MACE-free survival, followed by the *Warm and Wet* group. The *Cold and Dry* group exhibited an intermediate outcome between the *Warm and Dry* and *Cold and Wet* profiles. However, in the pairwise comparisons, significant differences were observed only between the *Warm and Dry* and *Warm and Wet* groups (p<0.01), and between the *Warm and Wet* and *Cold and Wet* groups (p=0.03). No significant difference was found between the *Warm and Dry* and *Cold and Dry* groups (ie, between the two *Dry* profiles) (p=0.23). When patients were stratified by sarcopenia status based on the skeletal muscle mass index (SMI <7.0 kg/m² for men and <5.7 kg/m² for women), the prognostic value of our profiling remained statistically significant within the non-sarcopenia subgroup (online supplemental figure S1), although it did not reach statistical significance within the sarcopenia subgroup.

## Discussion

In this study, we demonstrated the clinical potential of a virtual Forrester profiling that combines BIA with STI obtained from PWA. Specifically, among patients admitted to the cardiology ward, those classified as *Warm and Dry*—with both low ECW/TBW ratio and low STI—had a significantly lower risk of cardiovascular events compared with other groups.

Since the original Forrester classification was proposed in 1976, numerous less invasive and more practical alternatives have been suggested.^3 14^ Among them, echocardiography-based approaches are considered particularly feasible. For example, Takahashi *et al* calculated cardiac output and CI from the time-velocity integral of ejection flow obtained via pulsed-wave Doppler imaging of the left ventricular outflow tract, and estimated the presence of elevated left atrial pressure using the ratio of early diastolic trans-mitral flow velocity to early diastolic septal mitral annular velocity (E/e′).^4^ By classifying heart failure patients into four haemodynamic profiles based on these parameters, they showed that this non-invasive method could predict heart failure deterioration. However, as Garcia-Elcano *et al* recently reported, echocardiography still suffers from substantial inter-observer variability.^15^ Although performing focused scans using lung ultrasound or VEXUS scoring has been proposed as a potential solution, these approaches still require specialised technical skills and operator experience. In contrast, BIA and PWA offer highly reproducible measurements that are largely independent of the examiner. Moreover, PWA can be completed in approximately 5 min,^16^ and BIA requires a maximum of around 3 min, making these assessments faster than conventional echocardiography for routine clinical evaluation. This represents a clear advantage, particularly in hospital settings facing staff shortages or where overutilisation of resources is a concern.

Of course, both BIA and STI have unavoidable inherent limitations. Due to the principle of BIA, which measures body composition by passing a weak electrical current through the body, it cannot be applied to patients with pacemakers or other cardiac implantable electronic devices (CIEDs). In addition, in the type of scale-based device we used in this study, measurement is difficult in patients unable to maintain a stable posture for a sufficient period. Therefore, for estimating PAWP in patients with CIEDs or those who can only be examined in a sitting position, remote dielectric sensing may serve as a useful alternative.^17^ Furthermore, severe extracellular fluid overload can cause hyperhydration of skeletal muscle, which may lead to an overestimation of the SMI and potentially mask underlying sarcopenia or cardiac cachexia.

For STI, accurate calculation requires high-quality recordings of the ECG, PCG, and pulse wave. For instance, in patients with severe aortic stenosis, systolic murmurs and attenuation of the second heart sound (S2) can make it difficult to correctly identify the systolic period. Conversely, failure to obtain reliable STI measurements may serve as a clue to underlying structural heart disease, as abnormalities in the ECG or PCG could be detected. Alternatively, Piccirillo *et al* demonstrated that transthoracic bioimpedance allows for the non-invasive evaluation of haemodynamic parameters, such as stroke volume,^18^ which might offer a more feasible approach than STI in certain clinical settings. Similarly, Albert *et al* reported that bioimpedance-derived cardiac output showed a high correlation with the conventional thermodilution method, further supporting its clinical utility.^19^

One potential practical application of our risk assessment method is in the evaluation of cardiovascular risk before non-cardiac surgery. Tank *et al* reported that approximately 30% of patients undergoing abdominal surgery received inappropriate echocardiographic examinations.^20^ This is noteworthy because unnecessary preoperative echocardiography can sometimes be harmful.^21^ The 2024 **American College of Cardiology/American Heart Association** Guideline on Perioperative Cardiovascular Management recommends that preoperative echocardiography should only be ordered if the results are expected to alter management and explicitly states that routine preoperative echocardiography in asymptomatic, stable patients is not recommended.^22^ Therefore, a simple and quantitative risk assessment using BIA and STI could be beneficial as an alternative to overused echocardiography.

### Limitations

This study has several important limitations that should be acknowledged. Unlike BIA, which was performed in almost all patients as a standard assessment, PWA was ordered at the discretion of the attending physician. Consequently, the indications for PWA varied widely, including atherosclerosis screening, evaluation for peripheral artery disease and routine pre-catheterisation assessment as part of a clinical pathway. Therefore, selection bias cannot be excluded, and the applicability of our findings may be limited to certain patient populations.

Both BIA and PWA were typically performed on the day of admission or the day before diagnostic or therapeutic procedures. These time points may not be optimal for assessing the relationship between the measurements and post-discharge outcomes. Ideally, evaluations conducted just before discharge—after completion of treatment—would more accurately reflect long-term prognosis. This represents a major limitation inherent to the retrospective design of the study. Furthermore, the follow-up period varied across disease categories, and a substantial number of cases had observation periods shorter than 1 year. This variability limits the robustness of the survival analysis and should be addressed in the design of future prospective studies. Finally, in the receiver operating characteristic curve analysis, adding the Virtual Forrester matrix to BNP did not achieve a statistically significant improvement in predicting MACE (online supplemental figure S2). This lack of significance may stem from the strong pathophysiological correlation and overlap between ECW/TBW and BNP, as well as the inherent limitation of a time-insensitive binary analysis that fails to account for the precise time-to-event data. Although the combination matrix demonstrated a clear prognostic separation in the survival analysis, caution is required when applying these parameters in a cross-sectional risk-stratification framework without considering the temporal dimension of heart failure progression.

## Conclusion

The proposed virtual Forrester profiling integrating BIA and PWA provides a practical, quantitative and reproducible framework for non-invasive haemodynamic assessment, warranting validation in larger, prospective cohorts.

## Supplementary material

10.1136/openhrt-2026-004041 online supplemental file 1

## Data Availability

Data are available upon reasonable request.
