# Supplementary material for: Complementary cardiac functional assessment using systolic time intervals and bioelectrical impedance analysis
Source: Open Heart. 2026 Jun 8;13(1):e004041. doi: 10.1136/openhrt-2026-004041 (PMC13250219; doi:10.1136/openhrt-2026-004041)

Supplementary Figure S1.

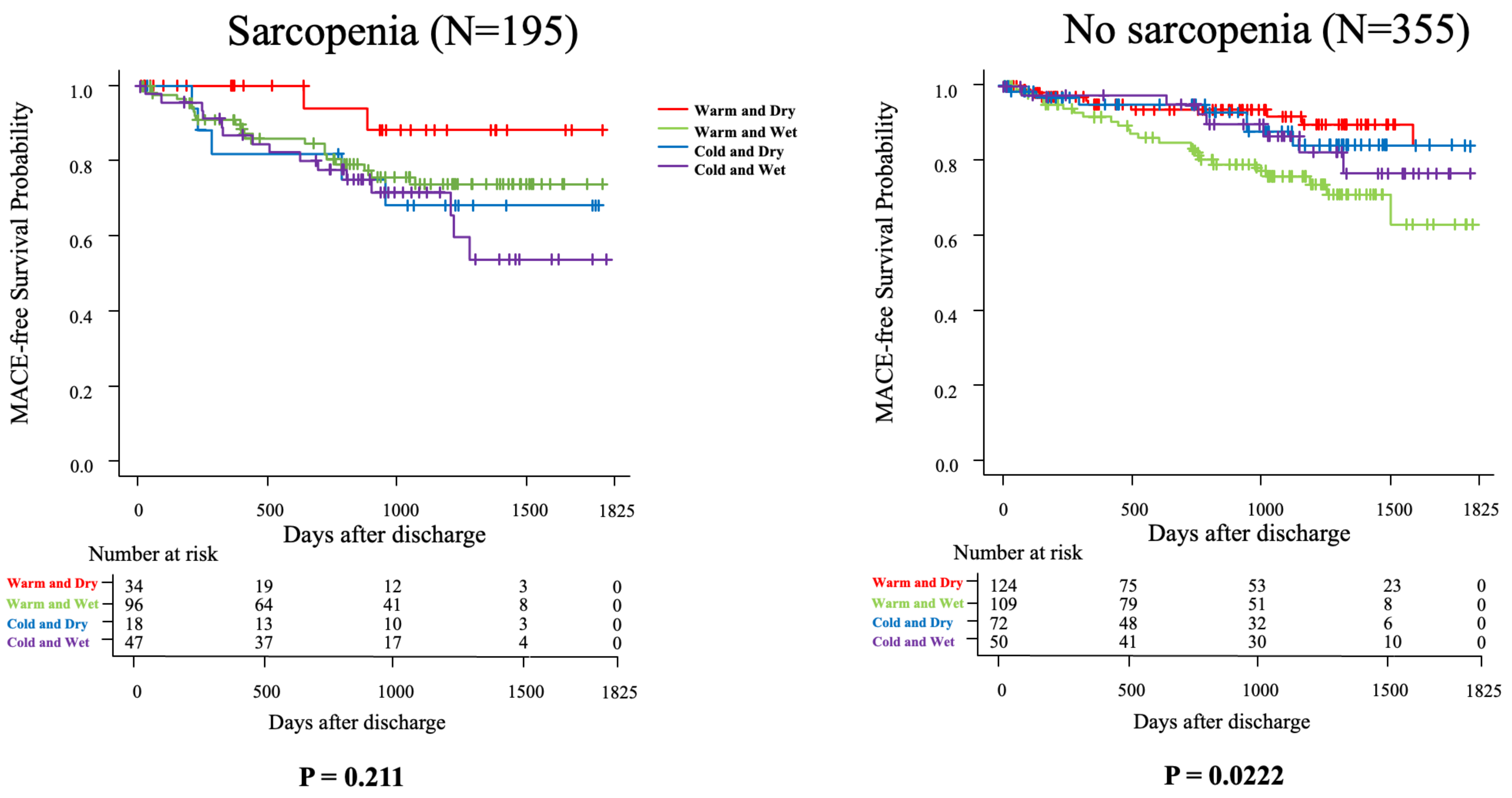

Supplementary Figure S2.

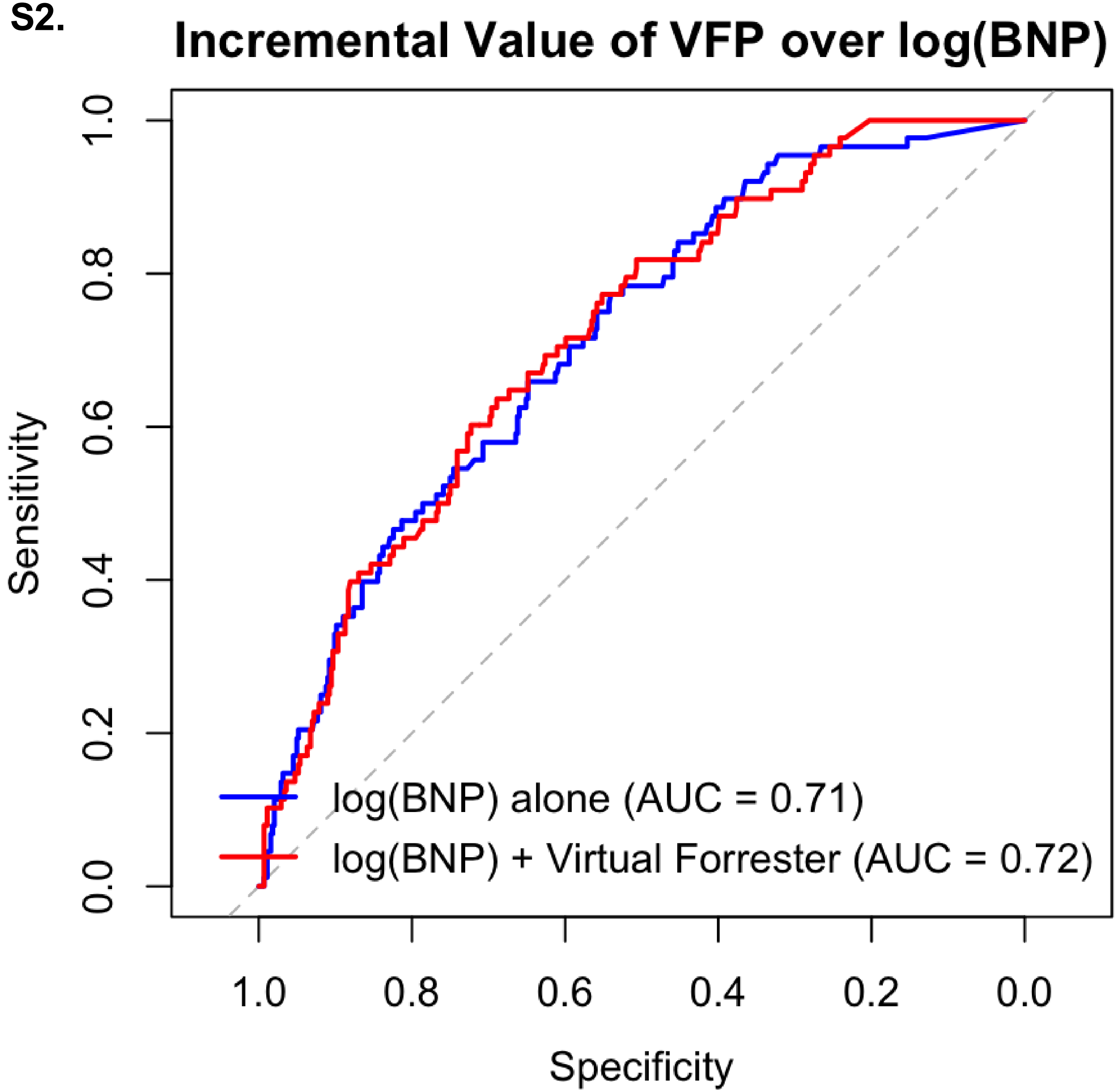

Supplement: online supplemental file 1 [file openhrt-13-1-s001.pdf]
